# Polymerization of chloro-p-xylylenes, quantum-chemical study

**DOI:** 10.1007/s00894-016-3179-6

**Published:** 2017-01-24

**Authors:** Cezary Czaplewski, Krzysztof Smalara, Artur Giełdoń, Maciej Bobrowski

**Affiliations:** 1 Department of Chemistry, University of Gdansk, Wita Stwosza 63, 80-308 Gdansk, Poland; 2 Department of Technical Physics and Applied Mathematics, Gdansk University of Technology, Narutowicza 11/12, 80-233 Gdansk, Poland

**Keywords:** Parylene, Chloro-p-xylylene, CVD, Polymerization, DFT, B3LYP, PBE0

## Abstract

The p-xylylene monomers of parylene N, C and D have similar high polymerization reactivity. For effective copolymerization processes this fact is basically a drawback and for instance the copolymerization with styrene doesn’t go at all (Corley et al. J Pol Sc 13(68):137–156, [15]). Substitution of terminal hydrogen atoms by chlorine atoms reduces reactivity dramatically. 7,7,8,8-tetrachloro-p-xylylene and 2,5,7,7,8,8-hexachloro-p-xylylene can be isolated as yellow crystals. These crystals can be kept without any change in temperature below 0 ^∘^C, but they polymerize slowly at room temperature. Perchloro-p-xylylene is stable even at elevated temperatures and does not polymerize under any conditions. Both 7,7,8,8-tetrachloro-p-xylylene and 2,5,7,7,8,8-hexachloro-p-xylylene copolymerize with various vinyl monomers, such as styrene and others. In this work the polymerization reactions of different chloro-derivatives of p-xylylene were modeled by means of the DFT method with hybrid correlation functionals (B3LYP and PBE0) and, for comparison, by means of the Hartree Fock methods. We inquired both initiation as well as elongation polymeric reactions for each of the reactants. We survied their reactivity analytically examining energetics and configurations in Szwarc-like process. The quantitative influence of chlorine atoms on the reactivity in polymerization steps, their location in the reactants’ structure (aromatic and/or aliphatic) as well as their number, were reviewed. The polymerizations of p-xylylenes with chlorine atoms as terminal aliphatic substituents yet revealed one more access path for parylenes’ in situ functionalization.

**Electronic supplementary material:**

The online version of this article (doi:10.1007/s00894-016-3179-6) contains supplementary material, which is available to authorized users.

## Introduction

**Fig. 1:**
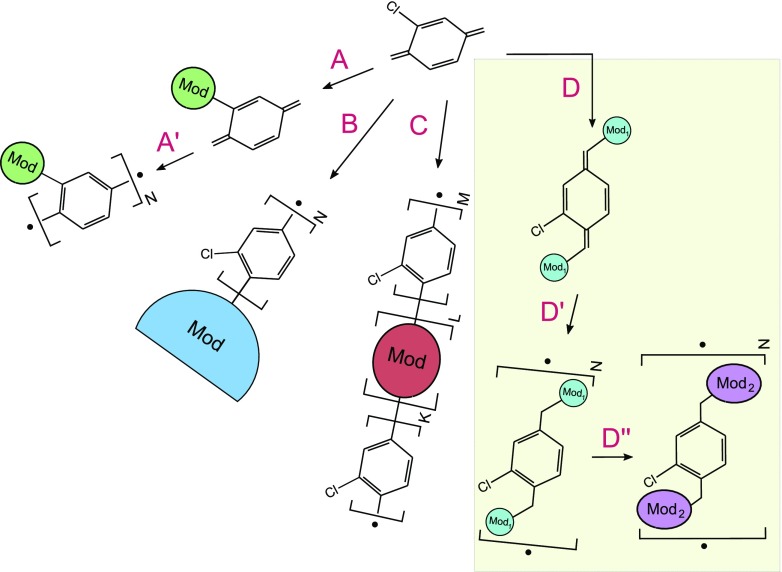
P-xylylene moieties can undergo various modifications to fabricate the desired CVD-produced parylene microstructure. The route A (and polymerization A$^{\prime }$) involves modified monomers and consequently leads to changing each parylene-chain unit. The route B requires the CVD process to occur in an active substrate, for instance in specific liquid-phase reactive alkenes. The resulting foil is expected to have one side chemically different than its second side, which means that after the functionalization the block structure of a parylene may further grow over the functional layer. The route C involves a gas-phase copolymerization between parylene monomers and other reactive monomers, namely like in the parylene-vinyl copolymerization. Relations between the K, L and M amounts depend basically on kinetics and molar ratios of both reactants. Routes D (and polymerization D$^{\prime }$) involve aliphatic substituents, for instance chlorine atoms. Relatively easy post-polymerization or in-situ aliphatic substitution (route D$^{\prime \prime }$) can guide through the next type of functionalization

Chemical vapor deposition (CVD) of [2,2]paracyclophanes and its analogues, which are commonly named parylenes, can have an effect on solids and on liquids [51]. Thin layers of parylene on solids possess almost perfect conformance to substrate topology while the liquids’ surface tension shapes the overgrowing polymer layer. The parylene layer is neither functional nor reactive, on the contrary it’s utilized as a passivation barrier protecting the devices from the environment such as chemicals or electric field. Not surprisingly the common applications of parylene cover rather those areas where the electrical insulation and chemical stability as well as biocompatibility play an important role [7, 20, 21, 29, 33, 43]. Parylene-C has been widely used as a biocompatible material for microfluidics and micro total analysis system (*μ*TAS) applications [9, 52]. However, its autofluorescence is an obstacle, since it is relatively high. On the contrary the parylene-HT exhibits low initial autofluorescence, decreasing autofluorescence behavior under UV excitation and higher UV stability, and can be a promising alternative for *μ*TAS applications with fluorescence detection [40]. Parylene-HT possesses aliphatic hydrogens saturated by fluorine atoms. Though it was found recently that the chemical deposition of parylene under reduced pressure over liquids which contain active vinyl molecules gains the chemical modification of parylene and most likely involves the double C = C bond of vinyls, as proved by FT-IR, FTRaman and XPS spectra. The same way of functionalization was accomplished by two separate groups in a relatively short time period and involved two different classes of molecules - unsaturated fluorenes [13] and (metha)acrylate-based moieties [42]. The experimental reports were followed by the theoretical description of the most likely corresponding reaction mechanisms [11, 22, 48]. Morevoer, even the kinetic studies were performed and followed by statistical analysis and the elegant information on the first-order structures of parylene-vinyl copolymers was achieved [16]. The on-the-fly functionalization methods seem to be competitive to the first-observed polymerization of functionalized [2.2]paracyclophanes with a wide variety of functional groups such as alkyl, carboxylic, hydroxyl, halogens, and amines which were consequently incorporated into the aromatic rings [14, 27, 34–38]. The second approach was used by McCarthy and Herrera-Alonso for aromatic electrophilic substitution accomplishing the direct chemical surface modification of parylene thin film [28]. There were also attempts to achieve oxidized surfaces with one attempt including plasma treatment [19, 41] and photo-oxidation [10, 45], (see Fig. 1).

The polymerization of [2,2]-paracyclophanes involves technologically p-xylylene molecule (the monomer) which originates from the cyclophanes after the pyrolysis in high temeperature. P-xylylene (3,6-Bis(methylene)-1,4-cyclohexadiene, also called p-quinodimethane) is a simple hydrocarbon molecule of both theoretical and commercial interest. Structurally it remains related to corresponding 1,4-benzoquinone by replacing the oxygen atoms with -CH _2_ groups. It is not stable in a solid or liquid form, however, certain substituted derivatives are stable, like tetracyanoquinodimethane (TCNQ) [2]. It was first discussed in the beginning of the 20th century by Thiele [51]. Thiele’s attempts to prepare the unsubstituted p-xylylene failed but he synthesized the isolable derivative of p-xylylene, *α*,*α*,*α*′,*α*′-tetraphenyl-p-xylylene, now often called a Thiele’s hydrocarbon. The very high reactivity of unsubstituted p-xylylene made its isolation very difficult and has precluded many experimental approaches to study this compound. It has been of interest of theoreticians even as a hypothetical compound [16]. In 1947 Szwarc showed evidence that p-xylylene was formed in the pyrolysis of p-xylene [49, 50]. The main product of rapid flow pyrolysis of p-xylene under reduced pressure is a white polymeric material, poly-p-xylylene. Members of poly(p-xylylene) family of polymers, including chlorine- substituted derivatives, become technically important materials, known as parylene polymers, when Gorham developed chemical vapor deposition process starting with the vacuum pyrolysis of di-p-xylylene ([2.2]-pcyclophane) [25]. This process is known as Gorham’s process and remains till today the leading one in parylenes’ fabrication. Essentially, the parylene material is an unreactive polymer, playing rather the mechanical barrier role, hence the many attempts to change this.

P-xylylene and its chlorine-substituted derivatives show very interesting differences in their chemical nature. The reactivity of chloro-substituted p-xylylenes decreases as the number of chlorine substituents is increased [30]. The p-xylylene is stable only in the gas phase or in a very dilute solution at a very low temperature. It is so reactive that it polymerizes spontaneously even at -78 ^∘^C. The substitution of one or two hydrogen atoms in an aromatic ring does not change reactivity and both 2-chloro-p-xylylene and 2,5-dichloro-p-xylylene polymerize spontaneously at low temperatures and cannot be isolated. Freshly deposited poly(p-xylylene) polymers contain a large amount of residual reactive free radicals. Based on electron spin resonance spectroscopy measurement the differences in apparent half-life of poly(p-xylylene) and of poly(2,5-dichlorop-xylylene) radicals were reported as 20 min and 21 h, respectively [47]. The substitution of terminal hydrogen atoms by chlorine atoms reduces reactivity dramatically. 7,7,8,8-tetrachloro-p-xylylene [23, 24] and 2,5,7,7,8,8-hexachloro-p-xylylene [31] can be isolated as yellow crystals. These crystals can be kept without any change in temperature below 0 ^∘^C, but they polymerize slowly at room temperature [31]. 2,3,5,6,7,7,8,8-octachloro-p-xylylene (perchloro-p-xylylene) [6] is stable even at elevated temperatures and does not polymerize under any conditions. A similar variation of monomer reactivity can be observed in their copolymerization reaction with vinyl compounds. No copolymerization of styrene or other vinyl monomer and p-xylylene was observed when they were passed together through the pyrolysis zone [15]. P-xylylene is too reactive for cross reaction with styrene [15]. On the other hand, both 7,7,8,8-tetrachloro-pxylylene and 2,5,7,7,8,8-hexachloro-p-xylylene copolymerize with various vinyl monomers such as styrene, vinyl acetate, acrylonitryle and methyl methacrylate [31]. The composition diagram of the copolymerization of 7,7,8,8-tetrachloro-p-xylylene and 2,5,7,7,8,8-hexachloro-p-xylylene with styrene shows that 2,5,7,7,8,8-hexachloro-p-xylylene copolymerizes better with styrene than 7,7,8,8-tetrachloro-p-xylylene does [31]. Monomer reactivity ratios based on copolymerization with styrene indicate that 7,7,8,8-tetrachloro-p-xylylene is more reactive than 2,5,7,7,8,8-hexachloro-p-xylylene. In the present contribution we apply quantum methods and try to digest reactivity of chloro-substituted xylylenes in polymerization reactions. Their behaviour in a deposition chamber and relative properties can lead to the formulation of competitive functionalization of parylene, especially where the aliphatic substituents can be relatively easy exchanged in nucleophilic substitution, theoretically even in post-processing the final (co)polymer. The facile tachnological process (the Gorham’s method) might remain the same or be only slightly modified.

## Methods

### Model substrates and reactions

**Fig. 2:**
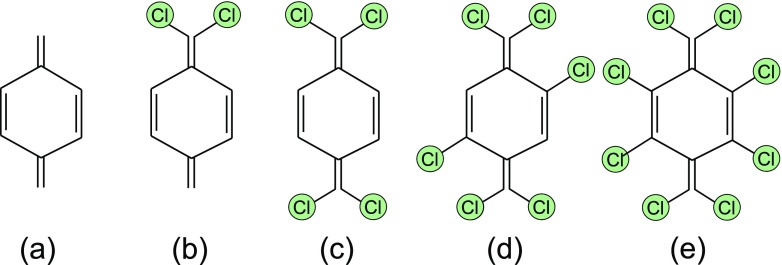
P-xylylene structure (**a**) and its chlorine derivatives: 7,7- dichloro-p-xylylene (**b**), 7,7,8,8-tetrachloro-p-xylylene (**c**), 2,5,7,7,8,8-hexachloro-p-xylylene (**d**), 2,3,5,6,7,7,8,8-octachloro-p-xylylene (perchloro-p-xylylene) (**e**) taken into account in the probe of polymerization reactions

In order to recount the quantitative influence of chlorine atoms on reactivity in the polymerization steps, their location in the reactants’ structure (aromatic and/or aliphatic) as well as their number, we shaped the following original p-xylylene-based reactants: (a) p-xylylene (for reference), (b) 7,7-dichloro-p-xylylene, (c) 7,7,8,8-tetrachloro-p-xylylene, (d) 2,5,7,7,8,8-hexachloro-p-xylylene, (e) 2,3,5,6,7,7,8,8-octachloro-p-xylylene (i.e. perchloro-p-xylylene). Their structures are outlined at Fig. 2. In the case of 7,7-dichloro-p-xylylene the initiation as well as further prolongation reactions can involve both aliphatic carbon atoms (*α*-carbons) with the chlorine atoms connected (atom no 7) or without the chlorine atoms (carbon atom no 8). Thus, we conducted separately both scenarios in the survey.

**Fig. 3:**
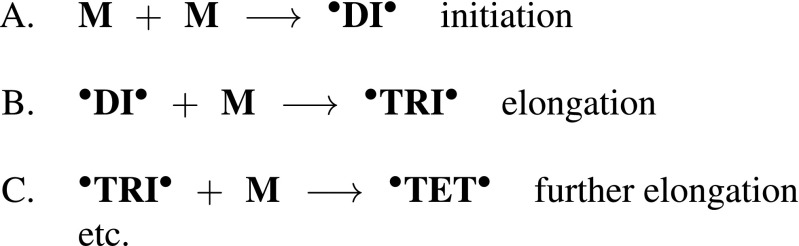
A stands for the initiation, B and C stand for the elongation (up to a tetramer). M stands for monomer substrates (i.e. closed-shell p-xylylene molecules and its chloro-derivatives), DI stands for dimer, TRI stands for trimer, TET stands for tetramer in open-shell configurations

We inquired into both initiation and elongation polymeric reactions for each of the reactants. The elongation reactions were continued up to tetramer, with the exception of 7,7-dichloro-p-xylylene where the elongation was paused at trimers. Step-growth polymerization usually requires substantial time to achieve good CVD-produced tiny parylene layer. Kinetic initiation and growth of polymer chains were first investigated by means of quantum methods which provided the extensive conformation and energy data. Energy profiles attained for each of the polymerization steps were then compared to each of the reactants, see Fig. 3.

### Quantum-chemical computations

The polymerization reactions of different chloro-derivatives of p-xylylene were modeled by means of the DFT Becke’s three-parameter hybrid method with the LYP (Lee-Yang-Parr) correlation functional (B3LYP) [8, 39]. Triple-zeta basis set with polarization functions (def2-TZVP) was used, as implemented in the Turbomole package software [5]. The advantage of DFT methods over post-Hartree-Fock methods is a smaller computational cost for dynamic correlation energy, especially for system sizes considered in this work (tetramer has 64 atoms). B3LYP functional has become a standard hybrid density functional used to study organic chemistry in the gas phase as it offers a good compromise between computational cost, coverage, and accuracy of results [46].

We also applied the PBE0 correlation functional [4] for almost all reaction steps investigated. The B3LYP and the PBE0 are both hybrid functionals as they contain a bit of the Hartree-Fock exchange energy, but the PBE0 does not contain any empirically fitted parameters and has 25 % of the Hartree Fock exchange (vs. 20 % for B3LYP) and is thus more ab-initio [53]. In other words, the PBE0 can be considered a true non-empirical DFT approach. A spin-unrestricted approach to time-dependent density functional theory was also successfully applied to small and medium-sized organic radicals [3].

In our previous work [48] we used independently the semiempirical CI AM1 and PM6 methods and the DFT B3LYP with two different basis sets to study polymerization steps of parylene N, C and D. It revealed that both semiempirical methods gave worse agreement with available experimental data compared to the DFT calculations, which can be most likely caused by the fact that parametrization of AM1 and PM6 methods is focused on closed-shell molecules.

Biradical molecules were treated as triplets with the UHF method. For all biradical molecules studied in this work the overlap between the open-shell orbitals is small and the unpaired electrons are located at different atomic centers. This fact allows for the description of open-shell biradicals using unrestricted density functional theory [26]. In the case of reactants and products of different multiplicities, the chemical reaction was modeled by using both standard restricted density functional theory (RDFT) and unrestricted density functional theory (UDFT). The transition state energy was estimated from the crossing between the two calculated potential energy profiles as in our earlier work [48].

When the spin symmetry breaking happens for a reaction then eigenstates are not eigenfunctions of 〈*S* ^2^〉. The wave function is said to be spin contaminated owing to the incorporation of higher spin state character as evidenced by expectation values of *S* ^2^ larger than *S*(*S*+1). DFT methods are not free from the problem of spin contamination too because of the presence of the Kohn-Sham orbitals. Basically, for the sake of structure optimization calculations one is limited to running an unrestricted calculation then projecting out the spin contamination after the wave function has been reached, which should lead to some amount of higher-spin states remove from the wave function and thus to the decreasing of the spin contamination. But this is only applied for the finally SCF-optimized wave functions. One rule of thumb which was derived from the experience with organic molecule calculations is that the spin contamination is negligible if the value of 〈*S* ^2^〉 differs less than 10 %.

We computed translational, rotational and vibrational contributions to partition functions of substrates and products (accordingly to the harmonic approximation approach) of reactions studied so it was possible to estimate the changes of the enthalpy (H), entropy and Gibbs free energy (G). The latter quantity is directly related to the equilibrium constant of a reaction. The rotational and vibrational partition functions and the respective contributions to H and G were computed following the Hessian calculation for each of the systems studied. We characterized in this way both transition states found and products of appropriate reactions.

For the cases where the multiplicity of reactants and product were the same, the transition states for each reaction were located by calculating the energy profiles of the reacting systems in internal coordinates most closely approximating the appropriate reaction coordinate, with the optimization of all remaining degrees of freedom. The geometry corresponding to the maximum in the energy curve was taken as an initial approximation to that of the transition state, and then the gradient norm was minimized to complete the search of this transition state. The Hessian matrix and subsequently the normal modes were calculated for all stationary points.

For transition states found we calculated the Global Electron Density Transfer (GEDT) based on the Natural BOnd (NBO) analysis in order to examine the charge nature of reactants and products in DFT B3LYP/PBE0 calculations and in the Hartree-Fock approach. This concept comes from the observation that the electron density transfer is not a local process in polar and ionic reactions but the charge transfer rather involves the global flux of electron density to electrophile [18]. For radical reactions, however, the flux might be relatively small and the GEDT indicating the charge transfer might show gas-phase polymerization reactions manifesting their radical nature. We also calculated the index values of new *σ*_*C*−*C*_ bonds for all reactions studied at the B3LYP level of theory in order to check whether new bonds were relatively advanced at various stages of polymerization and for diversely substituted chloro-p-xylylenes. This index is defined as follows: $l_{C-C} = 1 - \frac {r_{C-C}^{TS} - r_{C-C}^{P}}{r_{C-C}^{P}}$ where $r_{C-C}^{TS}$ stands for the C-C distance in the transition structure while the $r_{C-C}^{P}$ stands for the same distance in the corresponding product (dimer or oligomer) [32].

## Results and discussion

Below we first commented on polymerization reactions of parylenes N, C and D in the context of polymerization reactions of chloro-substituted p-xylylenes. We also demonstrated new results for the parylene N’s polymerization.

**Fig. 4:**
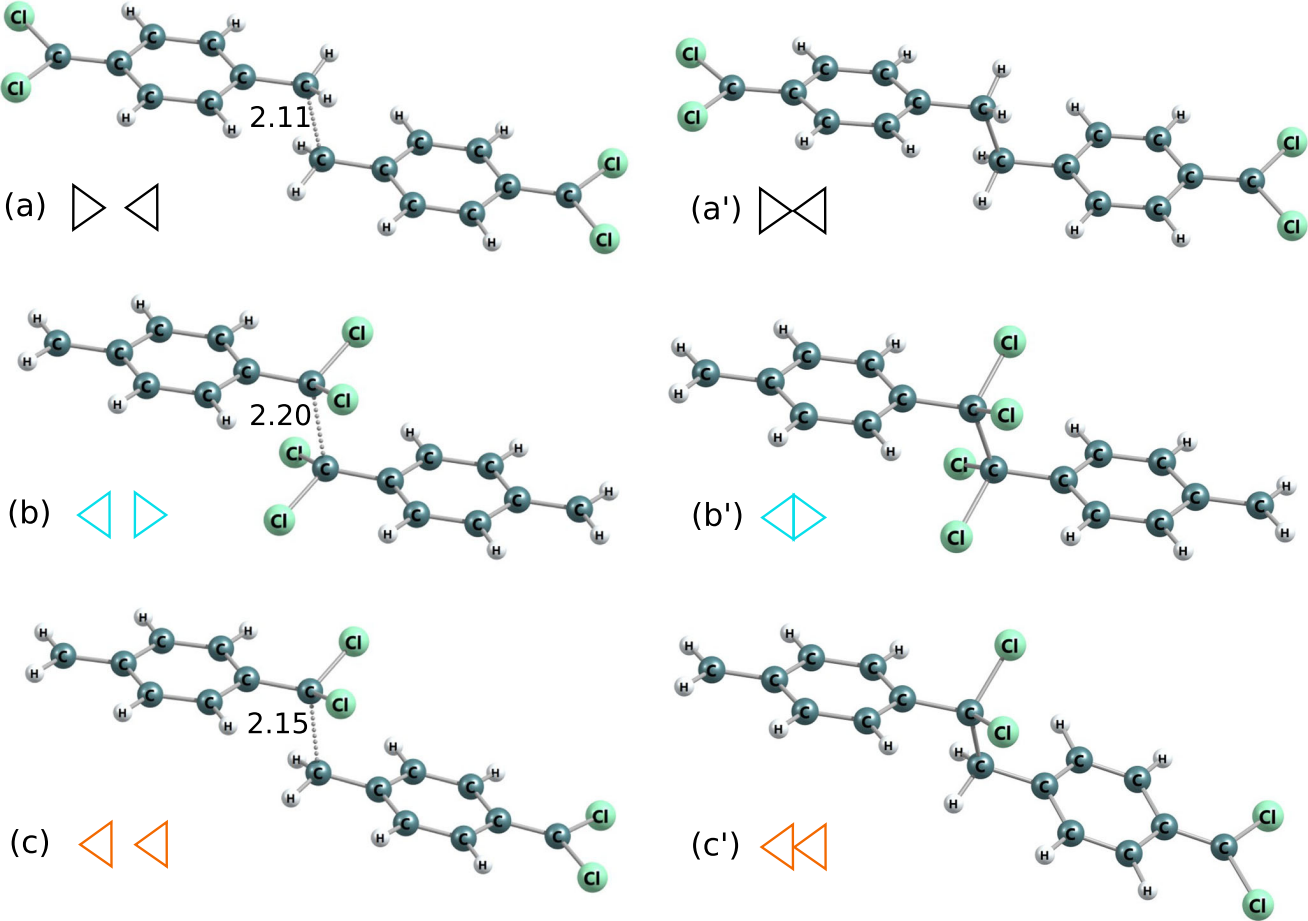
Molecular structures of DFT B3LYP-optimized dimers and corresponding most-close crossing-point configurations (for which C-C distances were shown) of polymerization reactions of 7,7-dichloro-p-xylylene. *Triangles* indicate the location of aliphatic -CCl _2_ groups as in Fig. 7 and as in Table 5

**Fig. 5:**
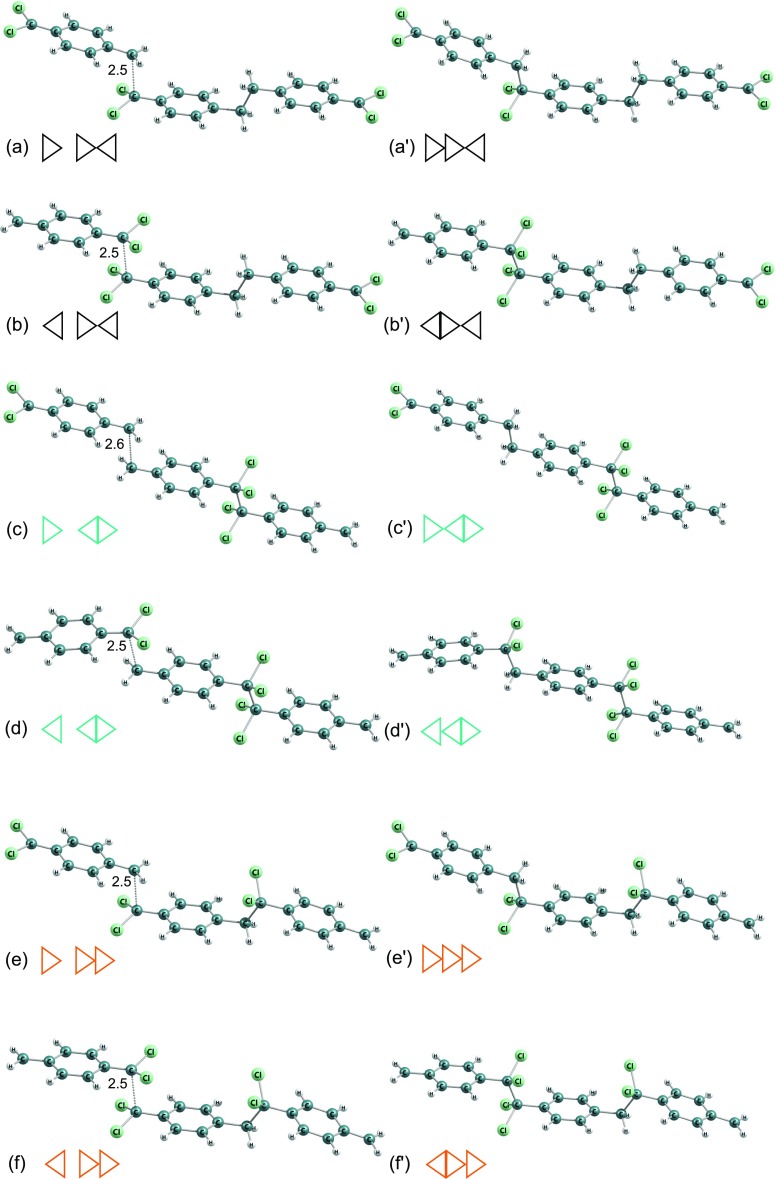
Molecular structures of DFT B3LYP-optimized trimers and corresponding saddle points of polymerization reactions of the 7,7-dichloro-p-xylylene. C-C distances were shown for saddle points. *Triangles* indicate the location of aliphatic -CCl _2_ groups as in Fig. 7 and as in Table 5

The lowest-energy DFT-calculated structures of monomers, dimers, trimers, tetramers as well as corresponding transition structures and most-close crossing- point configurations are depicted in Figs. 4, 5, 9, 10, 12, 13 and the energies are gathered in Tables 1, 4 and 5. The results put in the tables cover DFT B3LYP as well as DFT PBE0 calculations for all chemical paths investigated. Additionally, SCF-based Unrestricted Hartree- Fock computations were done also for the parylene N case for each stage of the polymerization (Tables 2 and 4).

**Table 1:** Relative energies [kcal/mol] (calculated against the energies of separated reactants, i.e. the singlet-state monomer and the triplet-state oligomer) revealed for all polymerization stages up to the tetramer of 7,7,8,8-tetrachloro-p-xylylene, 2,5,7,7,8,8-hexachloro-p-xylylene and 2,3,5,6,7,7,8,8-octachloro-p-xylylene

Method		Crossing	Dimer	Saddle	Trimer	Saddle	Tetramer
		M ⋯M	(Di)	Di ⋯M	(Tri)	Tri ⋯M	(Tet)
7,7,8,8-tetrachloro-p-xylylene	HF	25.75	−32.44	22.19	2.81	22.18	0.83
	〈*S* ^2^〉	3.11	3.24	4.31	3.24	4.80	4.23
	DFT B3LYP	25.79	5.10	11.57	−12.35	11.34	−12.55
	〈*S* ^2^〉	2.04	2.05	2.18	2.05	2.18	2.05
	DFT PBE0	19.76	−6.27	7.83	−21.08	7.79	−20.99
	〈*S* ^2^〉	2.06	2.07	2.26	2.07	2.26	2.07
2,5,7,7,8,8-hexachloro-p-xylylene	HF	24.79	−31.88	25.80	6.00	25.86	6.17
	〈*S* ^2^〉	3.27	3.39	4.53	4.00	5.13	4.60
	DFT B3LYP	28.10	8.59	13.90	−7.98	13.95	−7.92
	〈*S* ^2^〉	2.04	2.05	2.18	2.05	2.18	2.05
	DFT PBE0	21.63	−3.04	9.79	−16.79	9.94	−16.67
	〈*S* ^2^〉	2.05	2.07	2.26	2.07	2.26	2.07
2,3,5,6,7,7,8,8-octachloro-p-xylylene							
parall-parall	HF	60.03	19.59	41.76	26.83	41.70	26.62
	〈*S* ^2^〉	3.78	3.84	5.21	4.91	6.33	5.98
	DFT B3LYP	57.89	46.91	33.16	18.61	33.21	18.68
	〈*S* ^2^〉	2.09	2.10	2.13	2.10	2.13	2.10
	DFT PBE0	49.65	34.05	27.18	8.00	27.06	7.70
	〈*S* ^2^〉	2.12	2.13	2.19	2.13	2.20	2.13
parall-rot	HF	n/a	12.34	45.44	26.93	45.38	26.78
	〈*S* ^2^〉	n/a	3.75	5.21	4.81	6.28	5.89
	DFT B3LYP	n/a	34.70	39.36	18.74	39.48	18.85
	〈*S* ^2^〉	n/a	2.01	2.09	2.01	2.09	2.01
	DFT PBE0	n/a	21.35	34.08	8.59	33.92	8.33
	〈*S* ^2^〉	n/a	2.01	2.13	2.01	2.13	2.01
rot-rot	HF	n/a	16.06	41.68	26.75	41.74	26.77
	〈*S* ^2^〉	n/a	3.79	5.21	4.85	6.28	5.93
	DFT B3LYP	n/a	40.75	33.32	18.83	33.36	18.82
	〈*S* ^2^〉	n/a	2.05	2.09	2.05	2.09	2.05
	DFT PBE0	n/a	27.57	26.86	7.70	27.26	7.99
	〈*S* ^2^〉	n/a	2.07	2.13	2.07	2.13	2.07

Spin-square eigenvalues for each of the stages and for each of the theory level were shown. For the explanation of notations: parall-parall, parall-rot and rot-rot for the case of perchloro-p-xylylene see Fig. 13

**Table 2:** Thermodynamic potential-function and state-function changes: Δ*H*, Δ*G*, and Δ*S* calculated for DFT B3LYP harmonic contributions to partition functions of reactants, TSs and products (accordingly to the harmonic approximation approach)

	Transition state			Product		
	Δ*H*	Δ*G*	Δ*S*	Δ*H*	Δ*G*	Δ*S*
	kcal ⋅*mol* ^−1^	kcal ⋅*mol* ^−1^	cal ⋅*mol* ^−1^⋅*K* ^−1^	kcal ⋅*mol* ^−1^	kcal ⋅*mol* ^−1^	cal ⋅*mol* ^−1^⋅*K* ^−1^
p-xylylene						
DI	n/a	n/a	n/a	−11.00	1.11	−40.62
TRI	2.24	12.30	−33.72	−31.96	−18.29	−45.84
TET	1.78	8.66	−23.05	−32.01	−18.94	−43.83
7,7,8,8-tetrachloro-p-xylylene						
DI	n/a	n/a	n/a	5.54	20.93	−51.62
TRI	11.47	22.95	−38.51	−10.73	5.38	−54.03
TET	10.74	24.76	−47.04	−10.71	5.20	−53.36
2,5,7,7,8,8-hexachloro-p-xylylene						
DI	n/a	n/a	n/a	8.67	23.29	−49.02
TRI	12.27	30.05	−59.63	−6.90	8.97	−53.22
TET	12.88	27.81	−50.05	−6.63	11.60	−61.13
2,3,5,6,7,7,8,8-octachloro-p-xylylene						
parall-parall						
DI	n/a	n/a	n/a	61.95	45.82	−54.12
TRI	32.76	47.64	−49.92	19.33	37.01	−59.30
TET	32.69	45.67	−43.54	19.33	36.55	−57.73
parall-rot						
DI	n/a	n/a	n/a	34.45	49.25	−49.64
TRI	38.69	56.17	−58.62	19.25	36.47	−57.73
TET	38.75	55.96	−57.72	19.48	37.31	−59.79
rot-rot						
DI	n/a	n/a	n/a	40.07	55.59	−52.03
TRI	n/a	n/a	n/a	19.41	36.40	−57.01
TET	n/a	n/a	n/a	19.26	35.50	−54.47

The temperature was equal to 298.15 K and the pressure was equal to 1013 hPa (1 atm.). For explanation of notations: parall-parall, parall-rot and rot-rot for the case of perchloro-p-xylylene see Fig. 13

Also, for each level of theory, the spin contamination was evaluated by calculating the average value of square of the total spin operator - 〈*S* ^2^〉, see Tables 1 and 4. For each of chloro-substituted p-xylylenes we discussed it deeper below. Main TS’s geometry and other parameters were gathered in Table 3.

**Table 3:** TSs’ parameters: $l_{C-C} = 1 - \frac {r_{C-C}^{TS} - r_{C-C}^{P}}{r_{C-C}^{P}}$ where $r_{C-C}^{TS}$ stands for the C-C distance in the transition structure while the $r_{C-C}^{P}$ stands for the C-C same distance in the corresponding product (dimer or oligomer)

	*r*_*C*−*C*_ [ *Å*]	*l*_*C*−*C*_	GEDT			imag. freq. [cm ^−1^]		
	B3LYP	B3LYP	B3LYP	PBE0	HF	B3LYP	PBE0	HF
p-xylylene (parylene N)								
Di ⋯M	2.500	0.386	0.005	0.004	0.001	−316.02	−209.20	−462.31
Tri ⋯M	2.496	0.388	0.006	0.005	0.001	−326.63	−221.28	−459.94
7,7,8,8-tetrachloro-p-xylylene								
Di ⋯M	2.500	0.439	0.023	0.017	0.003	−421.88	−395.93	−538.82
Tri ⋯M	2.500	0.439	0.023	0.017	0.002	−420.98	−387.42	−540.77
2,5,7,7,8,8-hexachloro-p-xylylene								
Di ⋯M	2.500	0.448	0.023	0.017	0.003	−452.96	−421.88	−577.30
Tri ⋯M	2.500	0.446	0.025	0.018	0.003	−450.19	−428.71	−577.24
perchloro-p-xylylene								
Di ⋯M (parall-parall)	2.5999	0.410	0.001	0.003	0.001	−555.69	−571.84	−610.89
Di ⋯M (parall-rot)	2.5999	0.410	0.004	0.002	0.000	−482.18	−495.48	−603.94
Tri ⋯M (parall-parall)	2.3000	0.613	0.001	0.003	0.001	−556.23	−570.41	−609.93
Tri ⋯M (parall-rot)	2.3000	0.613	0.003	0.002	0.000	−493.26	−498.61	−591.90

GEDT stands for the Global Electron Density Transfer parameter. Imaginary frequencies were also shown. For explanation of notations: parall-parall, parall-rot and rot-rot for the case of perchloro-p-xylylene see Fig. 13

Additionally, in order to estimate the influence of thermodynamic conditions on the reactions’ feasibility following Hessian calculations we applied the harmonic approximation approach by calculating translational, rotational and vibrational contributions to partition functions of reactants, transition-state structures and products. The so-computed contributions allowed for the calculation of the thermodynamic potential- and state-function changes, i.e.: the Δ*H*, Δ*G*, and Δ*S*. The data is collected in Table 2 and the results are discussed below separately for each chloro-substituted p-xylylene. However, for each of the reactants they undergo the same type of reactions in the polymerization path while their structures remain relatively similar. It is therefore justified to predict that the entropy change might be comparable for each of the p-xylylenes cases. Instead, the energy factor might play the most important role in all cases of chloro-derivatives which will be discussed separately for each of the chloro-derivatives. In our previous work on polymerization reactions of parylenes N, C and D [48] we demonstrated that DFT B3LYP/TZVP computations reproduce the energetics of those reactions relatively well at each stage; the activation and elongation energies were in good (the best among all applied methods) agreement with the data available from the experiment. The same energy barriers found at semi-empirical CI AM1 and PM6 levels turned out to be much too high for those reactions. The relevant data was separately gathered in this work in Table 4.

**Table 4:** Relative energies [kcal/mol] (calculated against the energies of separated reactants, i.e. the singlet-state monomer and the triplet-state oligomer) revealed for polymerization stages up to the tetramer for parylene N

Method		Crossing	Dimer	Saddle	Trimer	Saddle	Tetramer
		M ⋯M	(Di)	Di ⋯M	(Tri)	Tri ⋯M	(Tet)
parylene N	HF	16.73	−47.18	9.62	−22.36	9.56	−22.42
	〈*S* ^2^〉	3.12	3.19	4.32	3.59	4.73	3.99
	DFT B3LYP	13.33	−12.52	3.37	−35.21	3.37	−35.22
	〈*S* ^2^〉	2.06	2.06	2.14	2.06	2.14	2.06
	DFT PBE0	9.25	−23.60	1.44	−43.75	1.18	−43.89
	〈*S* ^2^〉	2.08	2.09	2.19	2.09	2.20	2.09
(parylene N)	experiment	–	−14.2 ±5.0	–	–	–	–
parylene C	DFT B3LYP	14.89	−13.06	2.90	−35.81	3.02	−35.56
parylene D	DFT B3LYP	14.62	−13.28	2.46	−36.03	2.66	−35.96

The data for parylenes C and D were taken from the publication of Smalara et al. [48]. The experimental data of effect on the dimer formation comes from the publication of Pollack et al. [44]

Below, polymerization of each chloro-substituted p-xylylene is separately discussed and compared with the polymerization of other chloro-derivatives (2-chloro-p-xylylene forming parylene C and 2,5-dichloro-p-xylylene forming parylene D) and of p-xylylene (forming parylene N).

### Polymerization of parylenes: N, C, D

The parylenes N, C and D are currently the most widespread in industry p-xylylene polymers family. There are also other common halogenated parylenes, like parylene AF-4 or parylene HT (4 aliphatic fluorine atoms per one monomer), which can be recognized as an analog of Teflon because its aliphatic chemistry has the repeat unit -CF _2_- and as a result has superior oxidative and UV stability. The p-xylylene monomers of parylene C and D, 2-chloro-p-xylylene and 2,5-dichloro-p-xylylene, respectively, have similar polymerization reactivity [48]. However, they are heavier due to their molecular weight which influences higher threshold temperature and therefore a higher deposition rate, while still possessing a high degree of conformality. Substitution of one or two hydrogen atoms in main-chain aromatic rings has almost no influence on reactivity of the whole system. The so changed monomers can still polymerize in relatively low temperatures. On the other hand, such high reactivity of those polymers is basically a drawback in copolymerization processes, for instance with styrene [15]. This is caused by the fact that the vinyl and etin polymerizations are characterized generally by higher reaction barriers; many such processes require catalizers. Copolymerization of vinyl and parylene leads almost always (for various substituents present in vinyl moieties) to only insubstantial presence of vinyl units in the parylene main chains [12]. Hence the reveal that one possible unfolding for achievement of higher molar ratio for vinyl molecules in parylene-based copolymers might be the decrease of relative reactivity of parylene N, C and D’s monomers for instance by substitution of a larger amount of hydrogen atoms both in aromatic ring as well as in aliphatic segments.

Here we bring back shortly the reference of parylene N’s polymerization energetics for further comparisons with polymerization of chloro-derivatives designed in this paper and for which the experimental data were available. We also compare it with the data available for energetics of parylenes C and D. The data comes from the publication of Smalara et al. [48], where the results were achieved at the same (among others) DFT B3LYP/TZVP level. The experimental data comes from publication of Pollack et al. [44]. The reference results are collected in Table 4.

Additionally, we computed the same reaction paths by means of the DFT method but with the PBE0 hybrid functional which is more ab initio than the hybrid B3LYP [4, 53] within the unrestricted framework (for practical reasons DFT methods also use a DFT “wavefunction” constructed as an antisymmetrized product of one of the electron functions, defined as Kohn-Sham orbitals). And we did the same by means of the SCF-based UHF algorithm, yet estimating at all levels the spin contamination. The DFT PBE0 and the UHF results are also gathered in Table 4. As it turns out for each stage of the polymerization, the DFT PBE0 thermodynamic barrier is lower than those achieved after DFT B3LYP computations. UHF results do not lead to a similar conclusion, and the dimer seems to be almost twice as stable as after DFT calculations while the trimer and tetramer are almost twice as less stable then the trimer and tetramer, respectively, as achieved from DFT optimizations. The opposite situation exists for kinetic barriers, where the PBE0 functional gives lower energies then the B3LYP for all stages of polymerization. The UHF calculation in this case gives more similar results to those achieved at the B3LYP level but relatively higher, i.e. the saddle points are about 3 times higher in energy while the initiation reaction is almost 17 kcal/mol in comparison to about 13 kcal/mol found in B3LYP calculations. The spin contamination is large for the UHF wavefunction while for the Kohn-Sham functional the spin contamination is neglectable and for the triplet it is close to 2. DFT B3LYP results seem to be qualitatively the most close to experimental outcomes, while unrestricted Hartree-Fock calculations give rather feeble results.

As it reveals from the DFT B3LYP calculations, the kinetic barrier for reaction between two monomers is around 13 kcal/mol for p-xylylene, while it decreases by amount of around 1 kcal/mol for 2-chloro-p-xylylene and for 2,5-dichloro-p-xylylene. Here the influence of chloro atoms substituting hydrogens is only insubstantial. There is no experimental measurement for the activation barrier of this reaction. The computational data show that the reactions are exotermic with the biradical dimer having a lower energy compared to two monomers. This fact was also proven experimentally [44], where the energy effect of the biradical dimer formation starting from two monomers is around −14 kcal/mol, but this value has a substantial uncertainty.

The polymerization process indeed runs freely for the parylene family of polymers. A typical time necessary to produce a good-quality polymer layer over a liquid or solid substrate amounts to approximately 10 hours. The so-fabricated layers have their thickness ranging from 500 nm up to a few microns. The polymerization happens under 25 ^∘^C while the pressure is much lower than the atmospheric one. Probably this is the main reason why several kcal/mol for the dimerization is not a high barrier for the parylene-layer fabrication happening at a “room” temperature.

The data for the next reaction, between monomer (M) and biradical linear dimer (DI) forming biradical trimer (TRI), and between monomer and linear biradical trimer forming biradical tetramer reveal the elongation process is around 3 kcal/mol for all types of parylenes as found at the DFT B3LYP level. This is a much lower barrier than the barrier of forming cyclic dimer which is around 15 kcal/mol using the DFT method with the TZVP basis set. The difference between the energy barrier of the reaction of the two monomers and the monomer with the biradical dimer explains why growth of existing chain molecules is faster than forming a new chain, which must be initiated by creating the new linear biradical dimer. This, in turn, indicates that the whole polymerization process might involve column growth mechanism over the substrate [1].

In Table 2 we gathered the thermodynamic potential (Δ*H* and Δ*G*) and state (Δ*S*) function changes calculated for DFT B3LYP harmonic contributions to partition functions. We did calculations for typical “room” conditions i.e. for temperature equal to 298.15 K and the pressure equal to 1013 hPa (1 atm.). Δ*H* was found to be relatively small for transition states for parylene N and Δ*S* is negative, which even increases for the product of reactions (for the trimer and the tetramer (there is no thermodynamic data for crossing points for initiation reactions because the Hessian calculation would not make any sense)) as from two p-xylylene molecules we get one dimer. Comparing the Δ*S* for parylene N and the Δ*S* for chloro-derivatives one can conclude there is a tendency that the larger number of chlorine atoms in structures the larger Δ*S* calculated for each stage of the polymerization. The same pattern is observed for the energy factor, i.e. for kinetic energy barriers. Thermodynamic correlations will be discussed in more depth below for each chloro-derivative’s polymerization case.

### Polymerization of 7,7-dichloro-p-xylylene

This molecule has two different potential reactive *α*–carbons: in position 7 and in position 8 (on the opposite sides of the phenyl ring), see Fig. 2b. This escalates the potential number of possible structures of oligomers, because the monomer is antisymmetric in the oligomerization reactions. This fact leads to 3 different structures of dimers and 6 different possible structures of trimers, see Figs. 4 and 5. In Table 5 relative DFT B3LYP energies expressed in kcal/mol units are collected. The monomer structures are represented by triangles indicating the location of two aliphatic chlorine atoms in dichloro-p-xylylene, and in appropriate configuration of dimers and trimers as well as corresponding structures of saddle points and approximated energy crossing points of initiation reactions.

**Table 5:** DFT B3LYP/TZVP relative energies [kcal/mol] (calculated against energies of reactants) revealed for all polymerization stages of 7,7-dichloro-p-xylylene up to the trimer

	Crossing	Dimer		Saddle	Trimer
	M ⋯M	(DI)		Di ⋯M	(TRI)
	11.07	−17.52		−11.57	−46.51
				−5.15	−28.20
	28.77	9.64		12.24	−28.22
				17.66	−19.11
	18.85	−8.71		−2.83	−37.69
				3.92	−19.06

The triangles indicate location of aliphatic -CCl2 groups in 7,7-dichloro-p-xylylene, i.e. the  denotes the CCl _2_-Phe-CH _2_ monomer, while the  denotes the CH _2_-Phe-CCl _2_ monomer. The colors discern opening configurations. See also Fig. 7

**Fig. 6:**
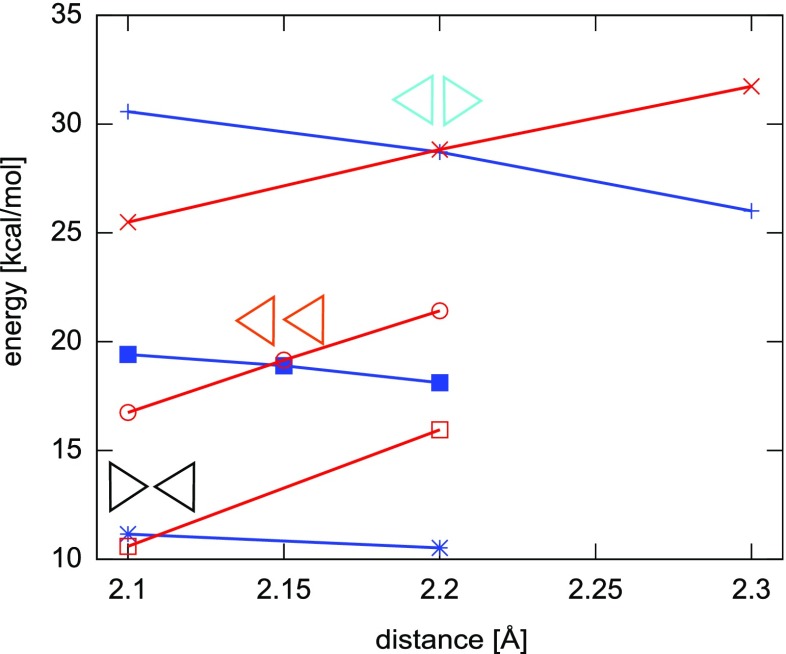
Crossings of energy profiles for the initiation reaction of dimerization of two 7,7-dichloro-p-xylylene molecules in different configurations calculated using the closed shell model (RHF, *blue color*) and the open shell model (UHF, *red color*) with the B3LYP/TZVP method. *Triangles* illustrate the location of the aliphatic -CCl _2_ group

**Fig. 7:**
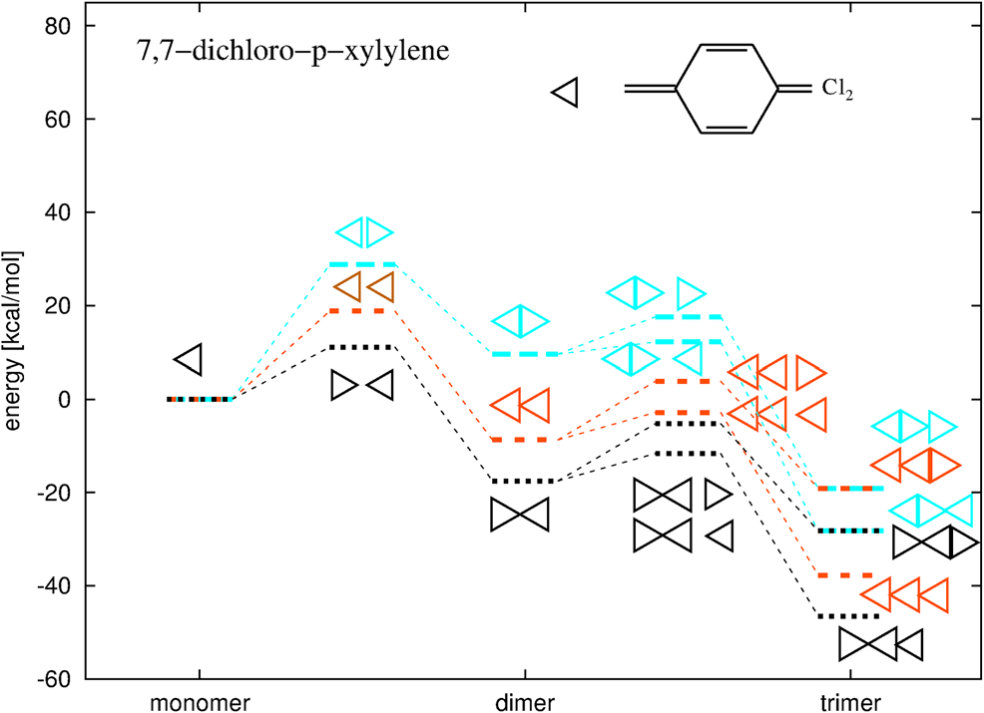
Energy diagrams for step-growth polymerization reactions of the 7,7-dichloro-p-xylylene up to trimers. *Triangles* illustrate the location of the aliphatic -CCl _2_ group

**Fig. 8:**
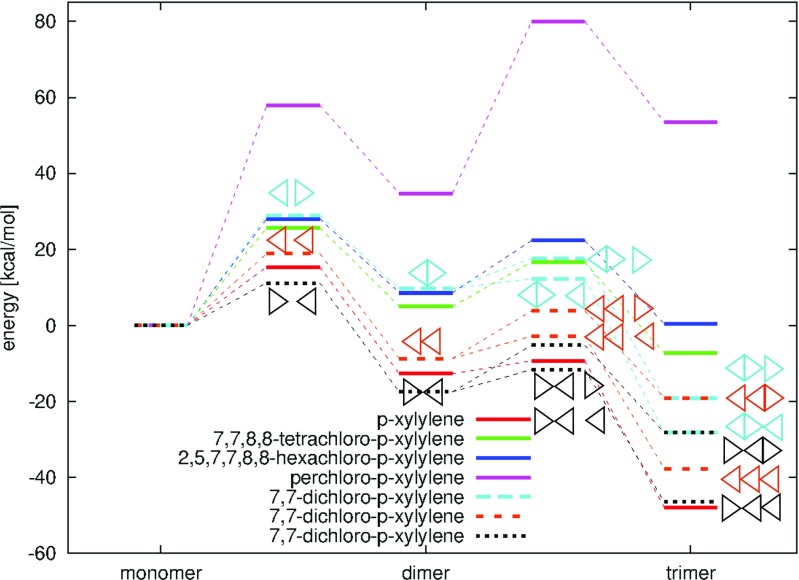
DFT B3LYP energy diagrams for step-growth polymerization reactions of the p-xylylene, the 7,7-dichloro-p-xylylene, the 7,7,8,8-tetrachloro-p-xylylene, the 2,5,7,7,8,8-hexachloro-p-xylylene and the perchloro-p-xylyene, up to the trimers. Triangles illustrate the location of the -CCl _2_ group in the 7,7-dichloro-p-xylylene and in respective oligomers

As it is revealed, the DFT B3LYP kinetic barriers of initiation reactions estimated by finding the crossing between two potential-energy curves for different spins, actively depend on configuration of atoms, i.e. on the mutual location of -CCl _2_ groups of two monomers: attachment of carbon 7 to carbon 7 (7–7) or of carbon 7 to carbon 8 (7–8) or even carbon 8 to carbon 8 (8–8). This is provoked by the strong electron repulsion of chlorine atoms which produces locally larger steric effect than hydrogen atoms do and where electron affinity is larger. This deduction seems to be proven by energy relations for the initiation reactions. The 7–7 reaction is approximately 9 kcal/mol more costly than the 7–8 and about 18 kcal/mol than the 8–8. Actually the 8–8 costs approximately the same amount of energy as the initiation reaction of polymerization of free-of-chlorines p-xylylene, see Table 4 and Fig. 8 for comparison. The initiation of 7–7 reaction is relatively highly energetic and the barrier is almost 30 kcal/mol which is much more in comparison to the initiation reaction of p-xylylene which energetic barrier amounts less than 14 kcal/mol [48]. The C-C distance for approximated crossing of energy profiles for the initiation reaction increases whenever the terminal carbon atom posses the chlorine atoms, see Fig. 6. Even the resulting biradical dimer is not so stable a molecule as opposed to 8–8 or 7–8 dimers. This situation repeats in further elongation reactions. The attachment of the next monomer to biradical dimer leads through first-order saddle point whose energy depends on the mutual orientation of -CH _2_ and/or -CCl _2_ of the dimers and attacking monomers. For all possible configurations the attachment of monomer involving its -CCl _2_ group is always less feasible in comparison to the attachment of -CH _2_ group of the attacking dichloro-p-xylylene by about 5 kcal/mol, see Fig. 7. What is also worth noticing, is the fact that the most stable are the trimers where the initiation involved the -CH _2_ groups of both monomers and the elongation involved also the -CH _2_ group of next (attacking) monomer. In contrast, the involvement of the -CCl _2_ group in elongation leads to less stable oligomers at any stage of the polymerization.

### Polymerization of 7,7,8,8-tetrachloro-p-xylylene

**Fig. 9:**
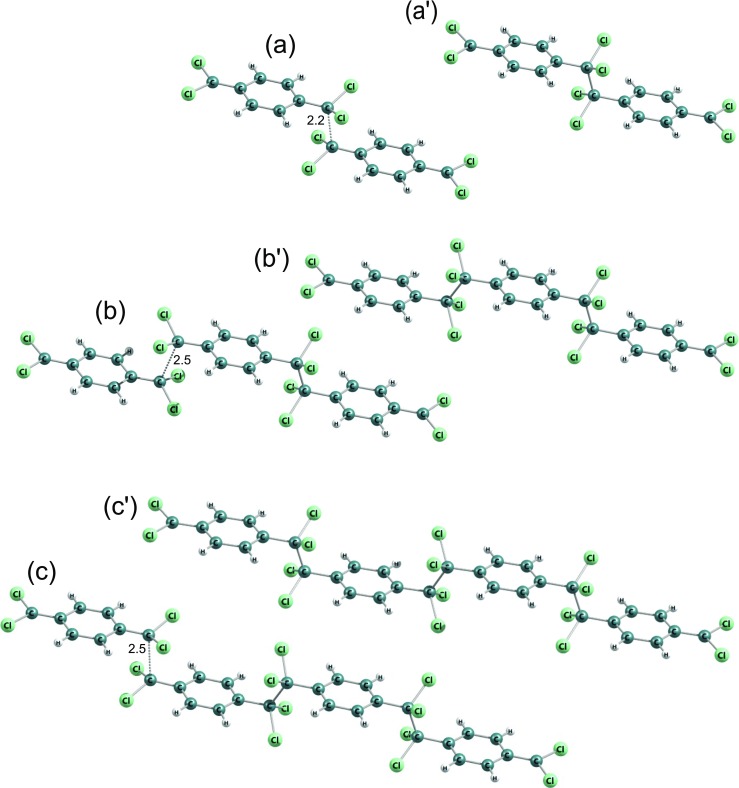
Molecular structures in DFT B3LYP-optimized stationary points of polymerization reactions of the 7,7,8,8-tetrachloro-p-xylylene: (a) to (a$^{\prime }$) - initiation, (b) to (b$^{\prime }$) and (c) to (c$\prime $) - elongation up to the trimer and up to the tetramer, respectively. C-C distances in respective saddle points (elongation reactions) and in energy-crossing points (the initiation reaction) were shown

The 7,7,8,8-tetrachloro-p-xylylene posses 4 aliphatic chlorine atoms (see Fig. 2c) which consequently stimulates lower reactivity in polymerization reactions in comparison to p-xylylenes with non-chlorinated aliphatic groups, see Figs. 8, 11 and Table 1. The optimized structures of resulting oligomers as well as the structures localized for transition states for elongation reactions and crossing energy curves for the initiation are shown in Fig. 9. The DFT B3LYP-calculated initiation reaction is almost 25 kcal/mol which is more than approximately 9 kcal/mol of the initiation energy for polymerization of reference p-xylylene [48] (see Table 4). The elongation costs approximately 10 kcal/mol against the optimized step-back oligomers and separated off monomer, at each stage of the polymerization studied up to tetramer. The presence of 4 aliphatic chlorine atoms causes relatively small increase of transition-state energies in comparison to polymerization energies of perchloro-p-xylylene, see Fig. 11. In the case of perchloro-p-xylylene there are also 4 aliphatic chlorine atoms and the polymerization also guides through the aliphatic carbon atom reactions. Thus, the presence of large amount of chlorine atoms (in aromatic locations) only increases significantly the energetic barriers and makes the oligomers less stable. On the other hand, the inclusion of two more chlorine atoms in the aromatic ring, like in the case of 2,5,7,7,8,8-hexachloro-p-xylylene (see Fig. 2d) yet makes the polymerization reactions feasible and the energetics seem to be very similar to the energetics of polymerization reactions of the 7,7,8,8-tetrachloro-p-xylylene (see Fig. 11) only slightly elevating the energies at each stage by amount of around 3 kcal/mol, as found on the basis of the DFT B3LYP calculations. As a consequence the symmetrical substitution of hydrogen atoms by chlorine atoms seems to be crucial in the decrease of feasibility of polymerization reactions of chloro-substituted p-xylylene’s derivatives. Similar effect has been found for the polymerization of parylenes: N, C and D [48], where the presence of chlorine atoms in aromatic positions in non-symmetric way does not elevate significantly the energetics.

As in the case of polymerization reactions of the hexachloro-p-xylylene the calculated DFT PBE0 energies remain smaller in comparison to the respective energies found in DFT B3LYP calculations, by an amount of 4 kcal/mol for saddle points at the paths to trimers and tetramers and by an amount of 6 kcal/mol for the initiation reaction. But the qualitative picture remains the same, i.e. the initiation reaction is much higher in energy then further elongation reactions. This is, however, different than it results from unrestricted Hartree-Fock calculations where the initiation costs approximately the same amount of energy as in the case of elongation reactions, see Table 1. For UHF-obtained wavefunctions, we found relatively large spin contaminations as the calculated mean values of squares of the total spin are large, especially for saddle points. For UDFT calculations respective 〈*S* ^2^〉 remains close to the value of 2 for triplets, which seems to be a typical observation for all DFT calculations for both B3LYP as well as PBE0 hybrid functionals and for all p-xylylenes studied.

Four chlorine atoms in aliphatic positions significantly change the feasibility of the polymerization of tetrachloro-p-xylylene in comparison to the feasibility of parylenes: N, C and D’ polymerization. This is also well noticeable after the examination of calculated enthalpies in saddle points and in products of reactions, see Table 2. Similar relations were found for other chloro-p-xylylenes studied. Calculated entropies are negative, as expected, because the attachment of monomers to radical oligomers always increases the order in the system. Typically, the calculated GEDTs, see Table 3, indicate for radical nature of polymerization reaction in the case of the tetrachloro-p-xylylene, similarly to other cases of p-xylylenes studied, with no charge transfer from the donor to the electrophile.

### Polymerization of 2,5,7,7,8,8-hexachloro-p-xylylene

**Fig. 10:**
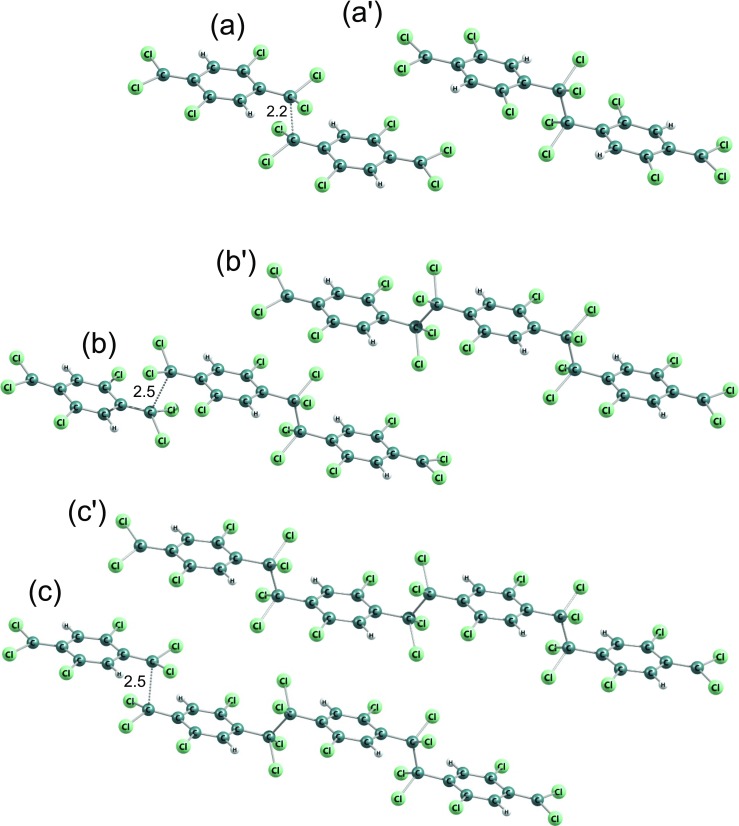
Molecular structures in DFT B3LYP-optimized stationary points of polymerization reactions of the 2,5,7,7,8,8-hexachloro-p-xylylene: (a) to (a$^{\prime }$) - initiation, (b) to (b$^{\prime }$) and (c) to (c$^{\prime }$) - elongation to the trimer and the tetramer, respectively. C-C distances in respective saddle points (elongation reactions) and in energy-crossing points (the initiation reaction) were shown

**Fig. 11:**
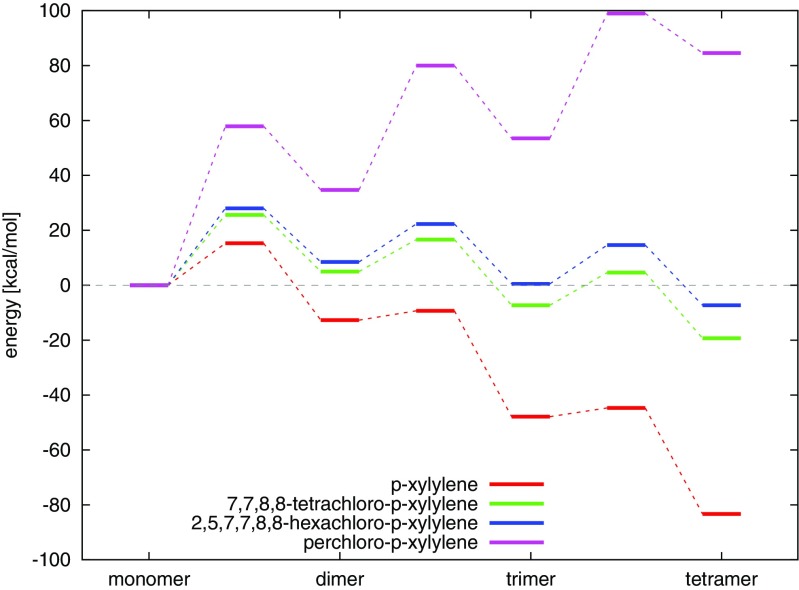
Energy diagrams for step-growth polymerization reactions of the 7,7,8,8-tetrachloro-p-xylylene, the 2,5,7,7,8,8-hexachloro-p-xylylene, the 2,3,5,6,7,7,8,8-octachloro-p-xylylene (perchloro-p-xylylene) up to tetramers. The energy diagram for polymerization reactions of the p-xylylene was also attached, for comparison

**Fig. 12:**
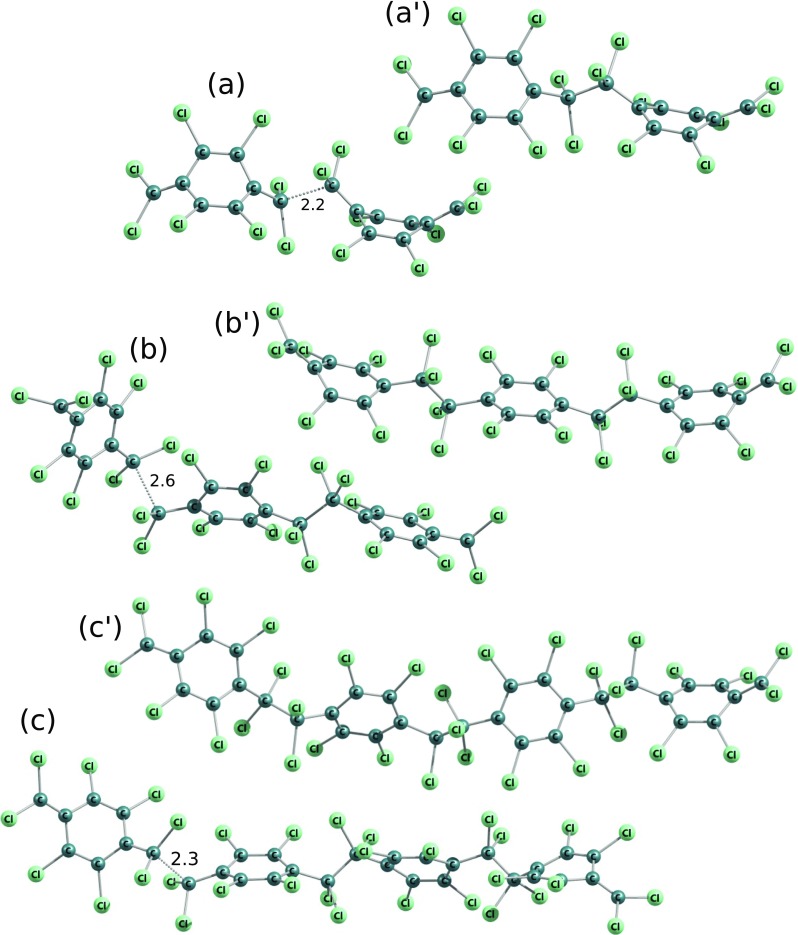
Molecular structures in DFT B3LYP-optimized stationary points of polymerization reactions of the 2,3,5,6,7,7,8,8-octachloro-p-xylylene: (a) to (a$^{\prime }$) - initiation, (b) to (b$^{\prime }$) and (c) to (c$^{\prime }$) - elongation to the trimer and the tetramer, respectively. C-C distances in respective saddle points (elongation reactions) and in energy-crossing points (the initiation reaction) were shown

DFT B3LYP and DFT PBE0 energetics of polymerization of the 2,5,7,7,8,8-hexachloro-p-xylylene is relatively similar to the respective energetics of polymerization of the 7,7,8,8-tetrachloro-p-xylylene, despite the fact that this molecule has two chlorine atoms more, see Table 1 and Fig. 11. On the other hand, the polymerization of this molecule is significantly different than the polymerization of 2,3,5,6,7,7,8,8-octachloro-p-xylylene (i.e. perchloro-p-xylylene) despite the fact that the perchloro-p-xylylene has only two chlorine atoms more and structurally aliphatic groups look the same. While the similarity to polymerization of 7,7,8,8-tetrachloro-p-xylylene is relatively comprehensible, the difference from the polymerization of perchloro-p-xylylene remains more thought-provoking. Looking at the Fig. 2d and e and at the Fig. 12 one can notice that in the case of perchloro-p-xylylene the aboriginal aromatic rings faintly lost their aromaticity and are no longer so plane. This means that in the case of symmetrical chlorine-substitution, the influence of electro-negative chlorine atoms in all hydrogen positions of aromatic ring (in positions 2 and 3 as well as in positions 5 and 6) is so siginificant and coordinated that their presence elevates the energies by 20 kcal/mol in the case of dimer, 40 kcal/mol for trimer and almost 90 kcal/mol for tetramer in comparison to respecitive energies of poly-(2,5,7,7,8,8-hexachloro-p-xylylene)’ oligomers, as found at the DFT B3LYP level. The DFT B3LYP kinetic barriers for the first three steps of 2,5,7,7,8,8-hexachloro-p-xylylene’s polymerization account for approximately 15 kcal/mol calculated against the energies of optimized step-back radical oligomers and separated monomer, only the initiation process costs almost 30 kcal/mol which is more than twice as much as the initiation process of parylene N’s polymerization. DFT PBE0 relative energies remain smaller, namely saddle points (at paths to the trimer and to the tetramer) have energies of approx. 10 kcal/mol lower than saddle points found at the DFT B3LYP level, while products of reactions have approx. 7–9 kcal/mol lower energies than those found by means of the B3LYP correlation functional. Comparing the same-levels-theory results, however, it turns out that the PBE0 gives about 2 kcal/mol higher-energy saddle points to appropriate products in the case of hexachloro-p-xylylene than in the case of the tetrachloro-p-xylylene. The opposite conclusion results for energies of dimers, trimers and tetramers, where they are approx. 3–4 kcal/mol higher for the hexachloro-p-xylylene’s polymerization in comparison to respective energies for the tetrachloro-p-xylylene. It is also conspicuous that the longer chains of poly-(2,5,7,7,8,8-hexachloro-p-xylylene) the more stable the chains are (see Fig. 11). Furthermore, the attachment of next monomers accounts for the elevation of relative energies of each corresponding step in comparison to polymerization’s energetic of very similar 7,7,8,8-tetrachloro-p-xylylene. Thus, one can conclude that the longer the oligomers, the larger influence of chlorine atoms on the stability of chloro-substituted poly(p-xylylenes). It also proves that in each following step of polymerization the resulting oligomer of poly-(2,5,7,7,8,8-hexachloro-p-xylylene) is less stable than the corresponding oligomer of poly(7,7,8,8-tetrachloro-p- xylylene). The optimized structures of all essential stages of 2,5,7,7,8,8-hexachloro-p-xylylene’s polymerization are collected in Fig. 10.

As in the case of other p-xylylenes studied in this work, UHF energies significantly deviate from energies found by means of DFT methods, see Table 1. For instance, the dimer seems to be unusually stable, while the trimer and the tetramer appear to be unstable, contrary to DFT results. But in the case of Hartree-Fock calculations the wave function is significantly spin-contaminated while in the case of DFT calculations the mean square of the total spin operator only negligibly varies from the value of 2 for triplets. For transition states found, calculated enthalpies are relatively higher than the respective enthalpies found for the tetrachloro-p-xylylene’s polymerization, analogously to calculated kinetic energy barriers which remain higher than the energy barriers found for the polymerization of tetrachloro-p-xylylene and the p-xylylene. Similar pattern can be observed for enthalpies of the perchloro-p-xylylene, but with the opposite relation. Calculated entropies are negative both in saddle point configurations and in products, and their absolute values remain similar in comparison to those found for other chloro-p-xylylenes, and are larger in the case of polymerization for parylene N, see Table 2. Calculated GEDTs in the case of the hexachloro-p-xylylene indicate that polymerization reactions do not involve charge transfers and remain of a radical type, as in the case of all other p-xylylenes studied, see Table 3.

### Polymerization of perchloro-p-xylylene

In the case of perchloro-p-xylylene moiety the polymerization’s energetics significantly deviates from the energetics of other chloro-substituted p-xylylene molecules. As it is known from experiments [6], the 2,3,5,6,7,7,8,8-octachloro-p-xylylene is stable even in relatively high temperatures and does not polymerize at all. As it is shown in Table 1 the hypothetical polymerization reaction would be very endoenergetic at all the possible stages of polymerization: initiation and elongation. The initiation barrier process is almost 60 kcal/mol and is thus almost twice as expensive as corresponding initiation reactions of 7,7,8,8-tetrachloro-p-xylylene and 2,5,7,7,8,8-hexachloro-p-xylylenes. What is interesting, in each elongation step the relative energy conforming the kinetic barrier of attachment of the next monomer to the existing biradical oligomer remains at the same level of approximately 40 kcal/mol calculated against the energy of the shorter open-shell oligomer and the closed shell monomer. As it is shown in Fig. 11 the oligomers of perchloro-p-xylylene remain very unstable despite the fact that the chains become longer after each step of the elongation process (in all other cases of modeled reactions of polymerization of chloro-substituted p-xylylenes the elongation of the chains makes the oligomers more and more stable). The molecular structures of DFT-optimized local minimas and corresponding transition states of each examined polymerization steps are shown in Fig. 12. The molecules are significantly different than analogous oligomers of other chloro-substituted derivatives of p-xylylene studied in this work; they are meaningfully deformed. It is visible that even the aromaticity of phenyl rings is a little lost as the rings are no longer so plane as they remain in the case of other chloro-substituted derivatives. Also the energy-minimized structures of oligomers expose that the chlorine substituents look not so stable, bent in comparison to analogous oligomers of other derivatives of p-xylylene, see Figs. 4, 5, 9, 10 and 12. The perchloro-p-xylylene seems to be a pole for the p-xylylene molecule. It contains all hydrogen atoms substituted by chlorines, both aromatic and aliphatic. From all chloro-derivative monomers this molecule seems to be the one whose polymerization is unlikely, in opposition to p-xylylene which polymerizes easy at room temperatures. The comparison with the polymerization of 7,7,8,8-tetrachloro-p-xylylene reveals that while the substitution of all aliphatic hydrogen atoms is yet attractive for the polymerization as the appropriate energy barriers amount several kcal/mol against the optimized step-back oligomers, the full chlorination effectively inhibits polymeric reactivity. Even the presence of two more chlorine atoms in aromatic positions, like in the case of 2,5,7,7,8,8-hexachloro-p-xylylenes does not stimulate much change in comparison to 7,7,8,8-tetrachloro-p-xylylenes, because the relative energies are only subtly changed, see Fig. 11. This finding leads to the conclusion that electro-negative chlorine atoms must work symmetrically to drastically change reactivity in polymerization reactions, yet leading to aromaticity’s loss of the benzene rings. It also indicates that there is no linear dependence in the relation between the number of chloro-substituents and the loss of polymerization reactivity.

**Fig. 13:**
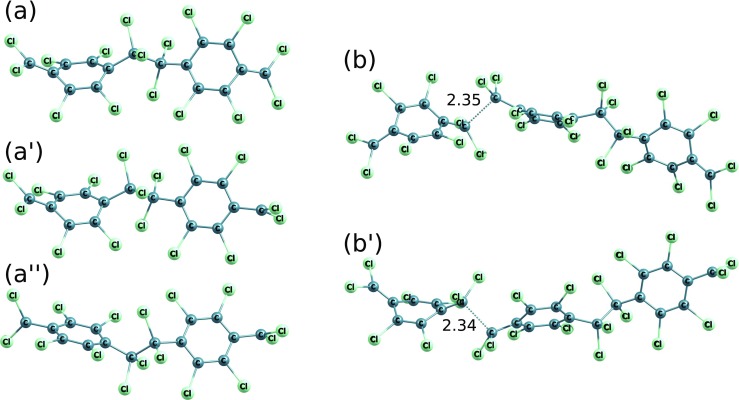
Molecular structures in DFT B3LYP-optimized dimers and transition-state structures of trimerization of perchloro-p-xylylenes. 3 different peripheral -CCl _2_ group orientation (against the nearest aromatic rings’ orientation) combinations were shown in dimers: (a) - two “parallel”, (a$^{\prime }$) - one parallel and one approx. 90 degrees rotated and (a$^{\prime \prime }$) - two groups approx. 90 degrees rotated, and 2 in saddle points found in paths to trimers: (b) - two ,,parallel,, -CCl _2_ groups and (b$^{\prime }$) - one “parallel” and one group approx. 90 degrees rotated

We found that for the dimer there are possible 3 different conforming structures which differ in the peripheral - CCl _2_ orientation against the orientation of the nearest aromatic ring. Specifically, the -CCl _2_ might be close parallel with respect to the orientation of the closest phenyl ring or it might be approx. 90 degrees rotated, which in summary leads to 3 different dimers: the same parallel orientations which we call parall-parall, see Fig. 13a, one terminal -CCl _2_ group in the parallel alignment and one approx. Ninety degrees rotated which we call parall-rot, see Fig. 13a ^′^, as well as the conformation where the two peripheral -CCl _2_ groups lie in approx. 90 degrees rotated positions against their nearest aromatic rings, see Fig. 13a ^″^. Despite this fact, the reaction of two perchloro-p-xylylenes leads to the same structures in crossing of two curves for singlet and triplet systems. Further, when trimerization is concerned, there are also three possible conformations analogous to dimers but only two possible conformations in saddle points in the paths to trimers and they correspond to the parall-parall orientation and the parall-rot, i.e. the rot-rot saddle point cannot be achieved, see Fig. 13b and b ^′^.

As it results from Table 1, systematically only one conformation is characterized by the lowest energy, which is the parall-rot and the same correlation outcomes for each level of theory. As in the case of other chloro-substituted p-xylylenes and as in the case of the p-xylylene, the application of PBE0 gives in general lower relative energies than in the case of application of the B3LYP in DFT calculations. UHF calculations give much higher relative energies in comparison to DFT energies and are characterized by much higher spin contaminations for resulting wave functions than in the case of DFT calculations, as it analogously was found in cases of other chloro-derivatives of p-xylylene and in the case of the p-xylylene itself, see Table 1. But relations do not blur the general picture which remains same for all levels of theory, the perchloro-p-xylylene does not polymerize, despite the fact that there are discrepancies for different levels of theories (DFT vs Hartree-Fock).

In the case of perchloro-p-xylylene investigations, the comparison of DFT B3LYP-calculated enthalpies for each of the conformers in saddle points and those found for dimers, trimers and tetramers lead to the same conclusions as in the case of comparison of relative energies, see Tables 1 and 2. The calculated entropies are the largest among entropies found for all chloro-derivatives, which is consistent with the pattern that the larger amount of chlorine atoms in the structures the larger the entropy change both in saddle points and in products of reactions. As previously, in the case of enthalpy and entropy changes the general conclusion is that the perchloro-p-xylylene does not polymerize and that the entropy does not play an important role in the comparison to the energy factor. It results that the energy remains the most important factor in all investigations.

Calculated TSs’ parameters, similarly to cases of other p-xylylene derivatives, indicate that reactions are not of the ionic type and that the charge transfer does not occur (small values of GEDT). C-C distances in calculated saddle points are larger in the path to the trimer and lower in the path to the tetramer, in comparison to C-C distances found for saddle points for other chloro-derivatives, see Table 3.

## Conclusions

First steps of polymerization of four chloro-substituted p-xylylenes were analyzed by means of quantum-chemical methods and compared with the polymerization of typical parylenes. We surveyed their reactivity analytically examining energetics and configurations in Szwarc-like process. Polymerization reactions were modeled by means of the DFT hybrid method with two different correlation functionals: B3LYP and PBE0, which were successfully applied in other cases. Additionally, SCF-based Unrestricted Hartree-Fock calculations were performed for the same chemical routes, for comparison purposes. For optimized products and for transition states found the characterization of extremes was performed by calculating various geometry, energy and charge properties. The choice of chloro-substituted p-xylylenes for polymerization reactions was dictated by the absence of the collective polymerization of parylene and styrene [15] in spite of the fact that p-xylylenes and some alkenes indeed react with each other as it was proven by FT-IR, FT-Raman and XPS spectra [12, 13, 42]. On the other hand, the substitution of terminal hydrogen atoms (in aliphatic positions) by chlorine atoms in p-xylylene makes the copolymerization feasible and 7,7,8,8-tetrachloro-p-xylylene as well as 2,5,7,7,8,8-hexachloro-p-xylylene copolymerize well with styrene, vinyl acetate, acrylonitryle and methyl methacrylate as it has also been proven experimentally [31]. This is a new track for possible parylene functionalization, it seems. The p-xylylene monomers are relatively too reactive, even the polymerization of 2-chloro-p-xylylene (leading to parylene C) as well as of 2,5-dichloro-p-xylylene (leading to parylene D) remain equally difficult in copolymerization and the kinetic activation barriers are very similar to those of p-xylylene in its polymerization. It reveals that the presence of aliphatic chlorine atoms significantly manifest the change in feasibility of parylene-vinyl cross reactions. It is worth mentioning here that the CVD process of parylenes polymerization is very attractive for many applications whilst parylenes’ chemical inertness seems to be unwelcome in further development. Substitution in aliphatic -CH _2_ groups reverses this situation. The 7,7,8,8-tetrachloro-p-xylylene and the 2,5,7,7,8,8-hexachloro-p-xylylene polymerize much weaker than the analogous p-xylylene. Actually their energy profiles are similar but the longer chains are created the more stable are the polymers. The 7,7-dichloro-p-xylylene polymerizes much easier when only the -CH _2_ groups are engaged but the involvement of terminal -CCl _2_ 2 group(s) significantly elevates the barriers so that they remain very similar to barriers of polymerization of 7,7,8,8-tetrachloro-p-xylylene and of the 2,5,7,7,8,8-hexachloro-p-xylylene. It was proven that the presence of chlorine atoms in aromatic positions does not influence much the reactivity of p-xylylene, which was also observed for parylenes C and D. An exception to this is the hypothetical polymerization of perchloro-pxylylene not polymerizing at all which was also observed experimentally [6], in spite of the fact that it contains the same number of chlorine atoms in terminal aliphatic positions. In this case probably the power of symmetrical locus of electronegative chlorine atoms is so strong and cooperative that the conjugation of *π* bonds in p-xylylene as well as in aromatic rings after the initiation and further elongation reactions is disturbed. The resulting chains are significantly different than those related to all other cases, with mutually twisted/bent mers and noted lost of aromaticity of benzene rings.

The polymerizations of p-xylylenes with terminal aliphatic substituents which were here the chlorine atoms yet revealed one more access path for parylenes’ *in situ* functionalization. This might also be the post-processing nucleophilic substitution in aliphatic positions, see Fig. 1D ^″^. It is a well-known fact that such processes are relatively much more workable in opposition to potentially analogous aromatic substitution of modified p-xylylene, see Fig. 1 (A and A ^′^).

As far as the quality of methods applied is concerned, one may conclude that DFT B3LYP/TZVP computations reproduce the energetics of reactions at each of the stages relatively well. The activation and elongation energies were in good (the best among all applied methods) agreement with the data available from the experiment. The DFT PBE0/TZVP method seems to, however, work pretty well, while it gives basically lower values of relative energies. Large discrepancies are observed for the UHF energetics and even the qualitative picture remains different in comparison to DFT results. The Unrestricted Hartree-Fock method did not allow for distinguishing between initiation and elongation reactions for p-xylylenes, but UHF wavefunctions appeared to be significantly spin-contaminated, in contrary to Unrestricted DFT (B3LYP and PBE0) calculations for which the average value of square of the total spin operator is close to 2 for triplets.

The energy remains the most important factor for reaction paths. The calculated Δ*H* and Δ*E* as well as the Δ*S* of reactions indicate that the entropy for the same type of reactions in the polymerization path with similar structures engaged remains similar. As the calculated Global Electron Density transfers remain small, we remain convinced that polymerization reactions are indeed radical at each of the stages and that gas-phase processes do not involve polar and ionic structures, as it was previously found for polymerization reactions of parylenes: N, C and D [48].

Quantum calculations allowed for the basic characterization of four chloro-derivative p-xylylene’s polymerization reactions up to the tetramer. Those methods, unfortunately, do not allow for studying the dynamics of the LPCVD of parylenes over a solid or liquid substrate. The well-achieved energetics might even so be the input data for the MD-regime hybrid algorithm [17] for dynamically growing polymer layers and the resulting system evolution as well as its further bulk structure and properties investigations.

## Electronic supplementary material

Below is the link to the electronic supplementary material.

(CSH 290 bytes)
